# A Sustainable Alternative for Postharvest Disease Management and Phytopathogens Biocontrol in Fruit: Antagonistic Yeasts

**DOI:** 10.3390/plants10122641

**Published:** 2021-12-01

**Authors:** Luis G. Hernandez-Montiel, Samir Droby, Pablo Preciado-Rangel, Tomás Rivas-García, Ramsés R. González-Estrada, Porfirio Gutiérrez-Martínez, Graciela D. Ávila-Quezada

**Affiliations:** 1 Centro de Investigaciones Biológicas del Noroeste, Calle Instituto Politécnico Nacional 195, Col. Playa Palo de Santa Rita Sur, La Paz 23096, Mexico; 2 Department of Postharvest Science, Agricultural Research Organization, The Volcani Center, P.O. Box 15159, Rishon LeZion 7505101, Israel; samird@volcani.agri.gov.il; 3 Tecnológico Nacional de México, Instituto Tecnológico de Torreón, Carretera Torreón-San Pedro, Km 7.5, Ejido Ana, Torreón 27170, Mexico; pablo.pr@torreon.tecnm.mx; 4 Departamento de Sociología Rural, Universidad Autónoma Chapingo, Carr. Federal México-Texcoco, Km 38.5, San Diego 56230, Mexico; trivas@pg.cibnor.mx; 5 Tecnológico Nacional de México, Instituto Tecnológico de Tepic, Avenida Tecnológico 2595, Col. Lagos del Country, Tepic 63175, Mexico; ramgonzalez@ittepic.edu.mx (R.R.G.-E.); pgutierrez@ittepic.edu.mx (P.G.-M.); 6 Facultad de Ciencias Agrotecnológicas, Universidad Autónoma de Chihuahua, Escorza 900, Col. Centro, Chihuahua 31000, Mexico

**Keywords:** biocontrol, antagonistic mechanisms, OMIC sciences, patents

## Abstract

Postharvest diseases of fruits caused by phytopathogens cause losses up to 50% of global production. Phytopathogens control is performed with synthetic fungicides, but the application causes environmental contamination problems and human and animal health in addition to generating resistance. Yeasts are antagonist microorganisms that have been used in the last years as biocontrol agents and in sustainable postharvest disease management in fruits. Yeast application for biocontrol of phytopathogens has been an effective action worldwide. This review explores the sustainable use of yeasts in each continent, the main antagonistic mechanisms towards phytopathogens, their relationship with OMIC sciences, and patents at the world level that involve yeast-based-products for their biocontrol.

## 1. Introduction

Fruit is an important resource in human diet because of its contribution in vitamins, minerals, organic acids, fiber, among others [1]. Moreover, obesity, cardiovascular, cognitive, skin, eye, lung, and bone diseases could be prevented through regular fruit intake [2,3]. Nowadays, the consumer demands fruit with a high-quality standard, both in appearance and in nutritional content [4,5]. However, postharvest fruit quality is affected by various factors, especially fungal diseases [6], which decrease its organoleptic properties and cause significant losses during storage, affecting up to 25% of total production in industrialized countries and more than 50% in developing countries [7,8].

The main strategy to control fungal infections at the postharvest level in fruit is the application of synthetic fungicides [9]. Nevertheless, these products have negative effects on human, animal, and environmental health [10,11] and induce resistance in phytopathogens [12,13]. 

The rise of biotechnology in the last decade has made biocontrol one of the most studied sustainable alternatives in reducing postharvest diseases by using antagonistic microorganisms against phytopathogens [14,15], which is considered a viable alternative to synthetic fungicides [16]. Among the microorganisms, yeasts stand out for their antagonistic capacity, for example, they have certain characteristics, such as genetic stability, efficacy at low concentrations; control towards different phytopathogens [17]; simple nutritional requirements; survival under adverse environmental conditions; compatibility with other chemical and physical treatments; resistance to synthetic fungicides; and absence of pathogenicity towards the host [18,19]. Additionally, the yeasts do not produce metabolites potentially toxic to humans or animals and do not contaminate the environment [20,21,22].

In this review, we describe the use and applications of yeasts as biocontrol agents and its role in global sustainable postharvest disease management of fruits, including the characteristics of antagonist yeasts, their mechanisms of action, interaction with OMIC sciences, and future trends in their application.

## 2. Global Overview of the Use of Yeasts for Fruit Disease Biocontrol

Around the world, different yeast species have been evaluated for in vitro and in vivo control of postharvest fruit pathogens (Table 1). Although biocontrol commercial products for postharvest disease control have been developed, the search for new antagonists continues to allow the development of more effective biocontrol products that can be incorporated into crop sustainable management including fruits [23].

## 3. Mechanisms of Action of Antagonistic Yeast towards Fruit Fungal Phytopathogens

### 3.1. Competition for Space and Nutrients

Competition for nutrients and space has been suggested to be the major mechanism of action by which yeasts exert their antagonistic action in inhibiting pathogenic fungi. Yeasts consume the necessary nutrients for their colonization and growth faster than the pathogens resulting in inhibiting spore germination, reducing its growth and infection level and, thus, decreasing infection and diseases development [50,51]. In addition, the synthesis of inhibitory compounds in yeasts is increased by the absorption of nutrients in situ or ex situ, improving their ability to biocontrol plant diseases [52].

The carbon sources that yeast consume include glucose, maltose, fructose, melezitose, and lactose, among others [53]. The determination of the nutritional needs and adaptation to the host of each yeast are important for their capacity as an antagonist [54]. 

### 3.2. Killer Toxin

Killer toxins are often glycosylated proteins produced by yeast of different species and can disrupt specific cell wall components (β-1,3-d-glucans, β-1,6-d-glucans, mannoproteins, and chitin), which result in fungal cell death (Table 2) [55,56]. Killer toxins attach to the cell membrane where they interact with a secondary receptor that result in changes in cell membrane permeabilization, DNA synthesis inhibition, cell cycle disruption, and RNA fragmentation [57,58].

Genetic studies in *Saccharomyces cerevisiae* have shown that the ability to produce killer toxins is cytoplasmically inherited and related to the presence of double-stranded linear RNA (dsRNA) plasmids, which are then encapsulated, forming non-infectious virus-like particles (VLP) within the cell cytoplasm [66]. All killer toxins are produced under acidic conditions, and their activity decrease with the increase in pH and temperature of the medium in which they are found—an increase in these variables is sufficient for the yeasts to stop producing them [67,68]. 

### 3.3. Lytic Enzymes

**Figure 1:**
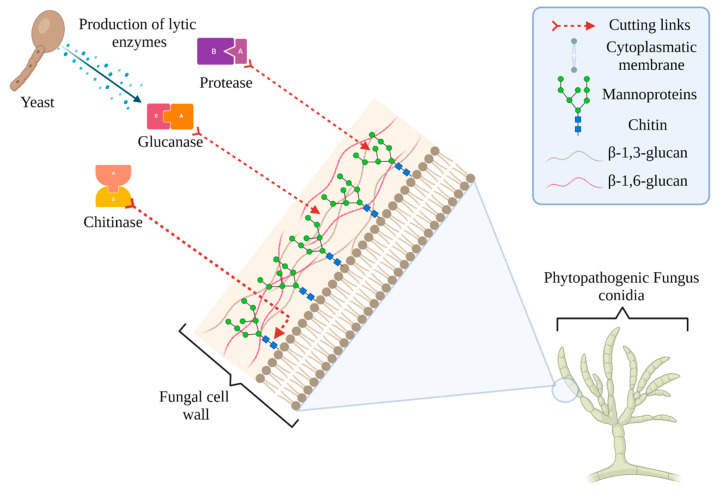
Enzyme production by antagonistic yeasts and their lytic effect on phytopathogenic fungus cell wall.

One of the antagonistic mechanisms of yeasts against phytopathogens is the production of lytic enzymes, such as glucanases, chitinases, and proteases, which act on different sites of the fungal cell wall, causing cell lysis and death (Figure 1) [69,70]. 

β-glucanases are enzymes that hydrolyze the β-glucosidic bond of β-glucans. There are two types of glucanases: those that randomly hydrolyze intra-chain bonds giving rise to oligosaccharides (endoglucanases) and those that release glucose molecules by hydrolyzing bonds at the non-reducing end of the chain (exoglucanase). There are also yeasts that can produce both types of enzymes [71,72]. Different mechanisms for glucanase synthesis and secretion have been suggested, but the most important one involves a synthesis regulated by repression in glucose when it is not found in sufficient quantities in the medium [73]. In relation to chitinases, these enzymes hydrolyze β-1,4 bonds of chitin *N*-acetyl-β-d-glycosamide, which is one of its main cellular fungus components, breaking it into oligomers and monomers of *N*-acetyl-β-d-glucosaminidase and causing cell death [74,75].

Five types of chitinases have been identified, of which the most common is type I with a molecular weight of around 30 kDa. In its sequence, it has an N-terminal domain similar to hevein and type II, which possess a lower molecular weight of 25 kDa and lack the N-terminal hevein domain [76,77]. Finally, proteases have a molecular weight of approximately 35 kDa, stability at a pH from 2 to 5, a low isoelectric point, are insensitive to metal and heavy metal chelators, and have a high capacity to hydrolyze a wide range of peptide bonds of the mannoproteins that make up the fungus cell wall [51,78].

### 3.4. Induction of Host Resistance

Yeasts can induce resistance in the host as an indirect mechanism to prevent infections caused by fungi [79]. At the initial stages of fungus invasion into the tissue, the fruit or plant cells begin with a hypersensitivity reaction (HR), which necrotizes the tissue invaded by the fungus to isolate the infection, to prevent, or to slow the advance towards healthy cells [80]. HR can be activated by many agents called inducers, such as synthetic products, phytopathogens, non-pathogenic microorganisms (such as yeasts, fungi, and bacteria), ultraviolet light, and insects, among others [81,82]. This reaction in the host can be systemic; due to this characteristic, induction is defined as systemic acquired resistance (SAR) [83]. 

In response to any inducer, the plant overexpresses genes and enzymes related to plant defense by increasing the production of substances. For example, these substances include proteins related to pathogenesis (PR-proteins, classified in 14 families) [84] and phytoalexins (characterized around 300, including coumarins, flavonoids, diterpenes, and benzofuran, among others) [85] and/or lytic enzymes (proteases, glucanases, and chitinases) [86] and reactive oxygen species (ROS) [87], among others, which have resulted in inhibition effects and/or cell lysis or disruption of the phytopathogenic fungus. 

PR proteins are defined as proteins that are absent or detected at a low basal level in healthy tissues but significantly accumulate during pathological conditions in both compatible and incompatible host–pathogen interactions [88]. Research studies involved PR-proteins following yeast treatment of fruit, i.e., *Pichia membranaefaciens* induced PR-9 and PR-10 in peach fruit [89]. However, PR-protein responses are too variable in relation to specific host tissue as well as microbial stimuli. The gene expression of PR-5 and PR-8 was characterized in apple fruit after treatment with *Candida oleophila* as a biocontrol agent against *Botrytis cinerea*. As a result, PR-8 was significantly overexpressed in response to both microorganisms while neither *B. cinerea* nor *C. oleophila* treatment significantly overexpressed the PR-5 gene [39].

Phytoalexin and lytic enzyme production by yeast resistance induction was demonstrated by Nantawanit et al. [90], who concluded that resistance induction in chili fruit treated with *Pichia guilliermondii* significantly enhanced the activities of phenylalanine ammonia-lyase (PAL), chitinase, and β-1,3-glucanase, and capsidiol phytoalexin accumulation in chili tissue. PAL is a fundamental enzyme during the first steps of the phenylpropanoid pathway to synthetize lignin, phenols, phytoalexins, and other compounds related to the plant resistance process [91].

Moreover, biocontrol yeast agents can enhance antioxidant enzyme activity to alleviate the oxidative damage caused by ROS produced in response to pathogen infection [65]. After cherry treatment with *P. membranaefaciens* at 5 × 10^7^ cells mL^−1^, peroxidase (POD) activity was enhanced, but catalase (CAT) and superoxide dismutase (SOD) decreased [92]. Many mechanisms related to resistance induction are simultaneously promoted by yeast antagonists. For example, *Rodosporidium* spp., *Pichia* spp., and *Cryptococcus laurentii* enhanced the activity of antioxidant enzymes and enzymes related to defense [93,94].

## 4. Antagonistic Yeasts and OMIC Sciences

Conventionally, the study of the mechanisms of action is related to the evaluation of the production of antibiotics, lytic enzymes, or other metabolites in vitro or in co-culture against the phytopathogen [95]. Information of the antagonistic mechanisms of antagonist yeasts is crucial for improving their efficiency against phytopathogens. Therefore, OMIC approaches, such as genomic, transcriptomic, and proteomic, are modern molecular technologies that help in their characterization [96]. Information on efficacy and consistency of an antagonist yeast helps to select the best antagonist against a specific phytopathogen [39]. The study of the microbial antagonist genome helps to understand the potential genes involved in biocontrol activity, characterizes groups of genes with unknown functions, compares the genome with other biological control agents, and, finally, helps in study gene transcription [97]. 

Proteomic approaches provide information on changes in metabolic/physiological functions within the cell. Additionally, any biotic or abiotic factors that induce changes during microbial growth can be studied by this molecular tool [98]. Proteomic analysis plays a key role in host–phytopathogen interactions, and this technique can help identify key proteins involved in antagonist–phytopathogen–host interaction [71]. 

Metabolomics analyses allow an understanding of cell physiology in real time. The production of secondary metabolites, antibiotics, and lytic enzymes is one of the main mechanisms of action for the control of phytopathogens [99]. The interaction of microbial antagonists with phytopathogens can change the proteome and transcriptome of plants or fruit, as well as their response to biotic stress through the induction of defense-related metabolic pathways [100]. 

Transcriptomic studies of biological control agents provide useful information on the genes involved in the production of secondary metabolites mostly studied in bacteria and yeasts [101]. In the case of fungal biocontrol agents, studies have focused on the genes involved in the influence of lytic enzymes, such as glucanases, proteases, and chitinases on fungal cell wall [102]. Transcriptomic analysis is not limited to the study of biological control agents; the study of phytopathogens also provides useful knowledge associated with its virulence [103]. 

Another important aspect is microbial interaction on the fruit’s surface with antagonistic microorganisms since they are an integral part of the host’s composition. The study of the microbiome is important to understand the key role of the microorganisms present and their role in fruit health and physiology, as well as their possible positive or negative interactions with artificially applied antagonists [104,105].

## 5. Patents on Yeast-Based Products for Plant and Fruit Disease Biocontrol

A patent, understood as the title that the state grants for the exclusive exploitation of an invention for a specified period [106,107], is a method used to protect intellectual property and, in many cases, is required to advance on the development of a biological product for the control of plant diseases. The first yeast-related patents date from 1842 in Finland [108] and 1873 in the United States by Luis Pasteur (US141972) [109]. 

**Figure 2:**
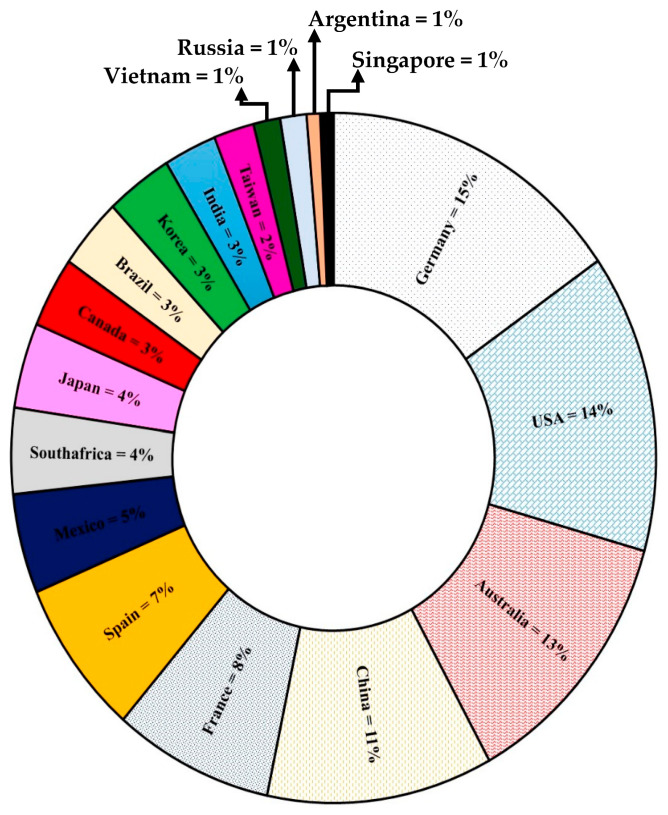
Percentage of patents by country of biocontrol products containing yeasts used in plants or plant parts.

Globally, from 2009 to 2021, 163 patents were reported in the Derwent Innovation database related to yeasts as biological control agents for plants or parts of them (Figure 2). Germany, USA, Australia, and China account for 53% of all patents with yeasts worldwide.

Of the 163 patents, 73.68% of the records have the name of the genus or genus and species of the yeast contained in the patented product, and 26.31% only indicated the word “yeast” among its components. Generally, the products contain yeasts and other microorganisms. Related to these products, 32.89% contained *Metschnikowia fructicola*; 11.18% contained *Candida* sp.; 11.18% contained a mixture of *Candida oleophila*, *Metschnikovia fructicola*, and *Pichia anomala*; 9.86% contained *Pichia* sp.; 7.89% contained *Rhodotorula* sp.; 5.92% contained *Cryptococcus* alone or mixed with *Rhodotorula* sp.; and 1.97% contained *Debaryomyces* sp. 

Moreover, 84.21% of the patents belong to companies, where Bayer^®^ is the predominant one. A low percentage is occupied by academic institutions (15.78%). This analysis reflects little participation of academics belonging to higher education institutions such as universities or public research centers in intellectual property registries. Much of the valuable information generated in universities has not been recorded, probably because the main objective is teaching and in addition to the lack of equipment to carry out mass formulations of the new product or an entity dedicated to marketing within these institutions. The development of biocontrol products containing yeasts for use in the post-harvest period is in high demand by entrepreneurs related to post-harvest and end consumer because it is a harmless product.

## 6. Conclusions

The use of yeasts as a post-harvest treatment to reduce decay caused by various phytopathogenic fungi in fruit of commercial interest is a sustainable and efficient alternative to the utilization of synthetic fungicides. The application of yeasts will be able to reduce the levels of fruit losses caused by phytopathogens, which will increase economic gains because of a greater volume of production for commercialization. Its implementation in postharvest will improve shelf life of the fruit and may lower crop costs by reducing the use of synthetic products. The acceptance of the consumer for product acquisition—not treated with any chemical—allowed opening new markets since it is a fruit not treated with synthetic fungicides.

## Figures and Tables

**Table 1:** Yeast antagonists evaluated for the biocontrol of postharvest diseases in five continents of the earth.

Continent	Yeast	Pathogen	Disease	Fruit	Inhibition Range (%)		Reference
					In Vitro	In Vivo	
**ASIA**							
China	*Candida oleophila*	*Botrytis cinerea*	Gray mold	Kiwifruit	-	17–45	[24]
Thailand	*Papiliotrema aspenensis*	*Colletotrichum gloeosporioides*	Anthracnose	Mango	66	94	[25]
India	*Candida tropicalis*	*Colletotrichum musae*	Anthracnose	Banana	70–85	84–96	[26]
Malaysia	*Trichosporon asahii*	*Colletotrichum gloeosporioides*	Anthracnose	Papaya	55–70	51	[27]
Israel	*Candida oleophila*	*Penicillium digitatum*	Green mold	Grapefruit	-	50–85	[28]
Taiwan	*Aureobasidium* sp.	*Botrytis cinerea*	Gray mold	Strawberry	18–36	-	[29]
Indonesia	*Aureobasidium* *pullulans*	*Colletotrichum acutatum*	Anthracnose	Chili	32–45	-	[30]
Saudi Arabia	*Pichia anomala*	*Botryodiplodia theobromae*	Fruit rot	Guava	-	39–50	[31]
**EUROPE**							
Italy	*Wickerhamomyces anomalus*	*Botrytis cinerea*	Gray mold	Strawberry	87	89	[32]
France	*Metschnikowia pulcherrima*	*Penicillium expansum*	Blue mold rot	Apple	52–91	15–18	[33]
Spain	*Hanseniaspora uvarum*	*Botrytis cinerea*	Gray mold	Strawberry	60–67	54–72	[22]
Poland	*Debaryomyces hansenii*	*Monilinia fructicola*	Brown rot	Apple	69	70–85	[34]
Germany	*Rhodosporidium paludigenum*	*Penicillium expansum*	Blue mold rot	Apple	-	67–86	[35]
Portugal	*Metschnikowia andauensis*	*Penicillium italicum*	Blue mold rot	Orange	62–70	90	[36]
**AMERICA**							
Uruguay	*Candida sake*	*Penicillium expansum*	Blue mold rot	Apple	25–74	25	[37]
Argentina	*Vishniacozyma victoriae*	*Botrytis cinerea*	Gray mold	Pear	-	70–100	[21]
Mexico	*Debaryomyces hansenii*	*Colletotrichum gloeosporioides*	Anthracnose	Papaya	15–36	66–83	[20]
Ecuador	*Candida inconspicua* and *Pichia kluyveri*	*Alternaria alternata*	Black rot	Pitahaya	-	7–20	[38]
	*Candida oleophila*	*Botrytis cinerea*	Gray mold	Apple			[39]
Brazil	*Aureobasidium* *pullulans*	*Penicillium digitatum*	Green mold	Citrus	30–41	-	[40]
Chile	*Crptococcus antarcticus*	*Botrytis cinerea, Penicillium expansum* and *Geotrichum candidum*	-	-	65–70	-	[41]
**AFRICA**							
Stellenbosch	*Pichia kluyveri*	*Botrytis cinerea* and *Monilinia laxa*	-	Apple	-	95–100	[42]
Tunisia	*Wickerhamomyces anomalus*	*Penicillium digitatum*	Green mold	Orange	100	100	[43]
South Africa	Various yeasts isolated from surface citrus fruits	*Penicillium digitatum*	Green mold	Citrus	-	95	[44]
Morocco	*Pichia anomala, Debaryomyces hansenii* and *Hanseniaspora guilliermondii*	*Penicillium digitatum*	Green mold	Citrus	-	65–80	[45]
**AUSTRALIA**							
Sidney	*Pichia guilliermondii*	*Botrytis cinerea, Alternaria alternata* and *Rhizopus nigricans*	Gray mold, black spot and *Rhizopus* rot	Cherry tomato fruit	-	25–90	[46]
Sidney	*Pichia guilliermondii*	*Colletotrichum acutatum*	Anthracnose	Loquat fruit	-	100	[47]
Sidney	*Cryptococcus laurentii*	*Botrytis cinerea*	Gray mold	Tomato fruit	-	55–90	[48]
Brisbane	*Rhodotorula glutinis*	*Penicillium expansum*	Blue mold	Pear	-	90–95	[49]

**Table 2:** Inhibition of phytopathogens cause of postharvest disease of fruits by yeasts producing killer toxins.

Killer Yeast	Phytopathogen	Fruit	Control (%)	Reference
*Debaryomyces hansenii*	*Alternaria brassicicola, Alternaria citri, Aspergillus niger* and *Rhizopus stolonifer*	Apple, tomato, and lemon	80–100	[59]
*Wickerhamomyces anomalus*	*Colletotrichum gloeosporioides*	Papaya	100	[60]
*Debaryomyces hansenii*	*Monilinia fructigena* and *M. fructicola*	Peach and plum	33–86	[61]
*Debaryomyces hansenii*	*Aspergillus niger*	-	80	[62]
*Pichia fermentans*	*Penicillium digitatum* and *P. italicum*	Lemon	40	[63]
*Wickerhamomyces anomalus* and *Meyerozyma guilliermondii*	*Colletotrichum gloeosporioides*	Papaya	20–24	[64]
*Saccharomyces cerevisiae and Wickerhamomyces anomalus*	*Penicillium digitatum*	Orange	87	[65]

## Data Availability

Not applicable.
